# Does active commuting improve psychological wellbeing? Longitudinal evidence from eighteen waves of the British Household Panel Survey

**DOI:** 10.1016/j.ypmed.2014.08.023

**Published:** 2014-12

**Authors:** Adam Martin, Yevgeniy Goryakin, Marc Suhrcke

**Affiliations:** a Health Economics Group, Norwich Medical School, University of East Anglia, Norwich, UK; b UKCRC Centre for Diet and Activity Research, Institute of Public Health, Cambridge, UK; c Centre for Health Economics, University of York, York, UK

**Keywords:** Active commuting, Physical activity, Wellbeing, Longitudinal study, Walking, Cycling, Health promotion

## Abstract

**Objective:**

The aim of this study is to explore the relationship between active travel and psychological wellbeing.

**Method:**

This study used data on 17,985 adult commuters in eighteen waves of the British Household Panel Survey (1991/2–2008/9). Fixed effects regression models were used to investigate how (i.) travel mode choice, (ii.) commuting time, and (iii.) switching to active travel impacted on overall psychological wellbeing and how (iv.) travel mode choice impacted on specific psychological symptoms included in the General Health Questionnaire.

**Results:**

After accounting for changes in individual-level socioeconomic characteristics and potential confounding variables relating to work, residence and health, significant associations were observed between overall psychological wellbeing (on a 36-point Likert scale) and (i.) active travel (0.185, 95% CI: 0.048 to 0.321) and public transport (0.195, 95% CI: 0.035 to 0.355) when compared to car travel, (ii.) time spent (per 10 minute change) walking (0.083, 95% CI: 0.003 to 0.163) and driving (− 0.033, 95% CI: − 0.064 to − 0.001), and (iii.) switching from car travel to active travel (0.479, 95% CI: 0.199 to 0.758). Active travel was also associated with reductions in the odds of experiencing two specific psychological symptoms when compared to car travel.

**Conclusion:**

The positive psychological wellbeing effects identified in this study should be considered in cost–benefit assessments of interventions seeking to promote active travel.

## Highlights

• Impact of commuting behaviour on wellbeing was explored using individual fixed effects analyses.
• Compared to driving, wellbeing was higher when using active travel or public transport.
• Use of active travel reduced the likelihood of two specific GHQ12 psychological symptoms.
• Switching from car driving to active travel improved wellbeing.
• Wellbeing increased with travel time for walkers, but decreased for drivers.

## Introduction

Regular, moderate-intensity physical activity can contribute to reductions in the risk of over twenty chronic health conditions (Biddle and Mutrie, 2007, Humphreys et al., 2014, World Health Organization, 2010). Whilst frequent physical activity is predictive of higher psychological wellbeing (Anokye et al., 2012, Bize et al., 2007, Cerin et al., 2009, Hamer et al., 2009, Teychenne et al., 2008), an increasingly important indicator used by Governments at the national level (Blanchflower and Oswald, 2004, Dolan et al., 2008, Office for National Statistics (ONS), 2013), only a small number of predominantly cross-sectional studies have specifically explored the impact of physical activity undertaken whilst travelling to work (Humphreys et al., 2013, Office for National Statistics (ONS), 2014, Roberts et al., 2011, St-Louis et al., 2014). Yet wellbeing could be an important, if often overlooked (Mokhtarian et al., 2001), component of utility (or satisfaction) measures that can be used in the cost–benefit analysis of transport policies (Powell et al., 2010) and in activity-based travel demand models (Ettema et al., 2010). These models focus not only on individual trips, where time savings alone are important, but seek to better understand how time is allocated across all trips and activities, allowing the impact on wellbeing of various interrelated factors such as travel patterns, urban form, and time use to be examined concurrently (Abou-Zeid and Ben-Akiva, 2012, Bhat and Koppelman, 1999, Bowman and Ben-Akiva, 2001, McFadden et al., 1977, Pinjari et al., 2011, Sallis et al., 2004).

Studies that examine the impact on wellbeing of active travel for recreational purposes, such as visiting friends (Hamer et al., 2009, Humphreys et al., 2013, Mutrie and Faulkner, 2004, Ravulaparthy et al., 2013, Teychenne et al., 2008), or as an intervention in clinical settings (Gusi et al., 2008, Stathopoulou et al., 2006), are more common than those that examine more routine active commuting. However, behaviour change in these non-work domains may be impractical for large numbers of working-aged people for whom the opportunity cost of physical activity outside of work hours is relatively high (House of Commons Health Committee, 2004, Martin et al., 2012, Popham and Mitchell, 2006).

In this paper, all 18 waves of the British Household Panel Survey (BHPS) (Taylor et al., 2001), a longitudinal survey of households in Great Britain, are used to study associations between wellbeing and (i.) travel mode choice, (ii.) changes in time spent commuting by specific travel modes and (iii.) switching to more active travel modes. As in comparable studies of wellbeing (Flint et al., 2013, White et al., 2013), including one by Roberts et al. that documented the predominantly negative associations with time spent commuting on the basis of analysis of the first 14 waves of the BHPS (Roberts et al., 2011), we used fixed effects (FE) panel data models which allow all unobserved factors that do not vary over time to be controlled. These can support more robust causal inferences than the previous cross-sectional studies (Humphreys et al., 2013, Office for National Statistics (ONS), 2014), which focused on statistical associations between wellbeing and time spent in active commuting and hence only contribute causal or interventional hypotheses (Bauman et al., 2002). With a particular focus on the impact of changes in individual-level travel behaviour on wellbeing, which could be more useful when assessing the case for behaviour change interventions, this study complements the already existing evidence on the physical health benefits of active commuting (Flint et al., 2014, Laverty et al., 2013, Wanner et al., 2012).

## Methods

### Data source and sample

The BHPS is a large-scale, multi-purpose longitudinal study of private households in Great Britain that began in 1991–1992 as an annual survey of each adult member of a nationally representative sample and ended after eighteen waves in 2008–2009. The sample used in the present study consisted of 17,985 adults aged 18–65 years who commuted to work.

### Variables

A 36-point Likert scale, increasing in psychological wellbeing (Goldberg and Williams, 1991), was used as the outcome variable in most analyses (hereafter, the 'GHQ12'). Twelve binary dependent variables were also created based on participants' ratings of the twelve specific psychological symptoms included in the 12-item General Health Questionnaire (Bowling, 2004, Goldberg and Williams, 1991), a widely used and validated instrument in patient and general populations (Hardy et al., 1999, McCabe et al., 1996), from which the Likert scale is derived (symptom present: ‘not at all’ or ‘same as usual’ = 0, ‘rather more’ or ‘much more than usual’ = 1, following Hamer et al.) (Hamer et al., 2009).

The primary exposures of interest were derived from the question “what usually is your main means of travel to work?” Two binary variables – for active travel (‘cycling’ or ‘walking’ = 1, other travel modes = 0) and public transport (‘train’ or ‘bus/coach’ = 1, other = 0) – and four mode-specific binary variables (e.g. ‘cycling’ = 1, other = 0) were created. The latter were also used to create interaction terms with commuting time and gender. Car travel (‘car/van’) was always included in the reference category. Remaining travel mode observations were excluded due to probable differential effects and/or small sample sizes: ‘car/van passenger’ (8.4% of total observations), ‘underground/metro’ (1.3%) or ‘motorcycle’ (1.0%) (although these were included in sensitivity analyses). In order to capture the impact of switching to a new travel mode, when compared to maintaining existing travel behaviour, binary ‘transition’ variables were created following Flint et al. if lagged (t − 1) and current (t) travel mode status were known (Flint et al., 2013). For example, to understand the specific impact of switching from car travel to active travel when compared to maintaining car travel, a transition variable was created where: ‘switched to active travel’ = 1 if ‘cycling’ or ‘walking’ in t and ‘car/van’ in t − 1; ‘maintained car travel’ = 0 if ‘car/van’ in t and t − 1; cases where lagged or current travel mode were unknown, or where other combinations of lagged and current travel mode were observed (e.g. switched from active to car travel, or maintained active travel), were excluded from the analysis.

The covariates included in the fully adjusted models, following Roberts et al., were: age squared, adjusted gross annual household income (four categories, accounting for size of household, including children's ages, using the McClements equivalence scale) (Taylor et al., 2001), number of children, self-assessed health status (three binary variables for ‘excellent’, ‘good’ and ‘fair’, each with ‘poor’ and ‘very poor’ in the reference category) (Dolan et al., 2008), educational attainment^✝^ (seven categories as in BHPS), work hours^✝^ (‘full-time’ = 1, ‘part-time’ = 0), neighbourhood characteristics^✝^ (binary variable derived from question: “Overall, do you like living in this neighbourhood?”), daily commuting time^✝^ (minutes) and job satisfaction^✝^ (1 = ‘completely dissatisfied’, to 7 = ‘completely satisfied’) (those marked^✝^ were excluded from the minimally adjusted models due to missing data being more common in these variables—see Results). Additional potential time-varying confounding variables were also included in the fully adjusted models: number of previous residences (= 1…n) and workplaces (= 1…n) (where n = number of residences or workplaces individual has reported since entering the sample, to account for house or job moves) (Booth and Van Ours, 2008, Clark et al., forthcoming, Dolan and Metcalf, 2008). Binary variables for each region and year were included in all models.

### Statistical analyses

The impact of change in the exposures of interest on a change in the outcome was assessed through variations within individuals over time using FE models. The benefit of using individual FE models is that they eliminate the risk that some time-invariant variables (e.g., some unobserved dimensions of socioeconomic status) may confound the relationship between travel mode choice and wellbeing. For example, current preferences for travel may have to a great extent developed in childhood/early adulthood, based on influences from parents and peers. Such influences may also continue to have some impact on the present sense of wellbeing. Given that influences that already happened earlier in life can be considered as fixed (and therefore time-invariant), individual FE models are ideally suited to deal with this sort of confounding. Hence causal inference is better supported using panel, rather than cross-sectional data (Baltagi, 2008, Wooldridge, 2010).

Table 1 provides a summary of four separate groups of analyses (subsequently referred to as ‘I’ to ‘IV’) that were completed using the following model specification:

(1) $$Y_{\mathrm{it}}\;=\;\alpha_{i}\;+\;\beta X_{k,\mathrm{it}}\;+\;\gamma Z_{j,\mathrm{it}}\;+\;u_{\mathrm{it}}$$

**Table 1:** Description of key features of four groups of analyses.

Features of the analysis	Four groups of analyses			
	I	II	III	IV
Dependent variable (Y_it_)	Psychological wellbeing^a^	Psychological wellbeing^a^	Psychological wellbeing^a^	Binary variable representing a specific psychological symptom

Main exposure of interest (X_k,it_)	Travel mode binary variable(s)	Commuting time-travel mode interaction terms	Travel mode transition variable(s)	Travel mode binary variable(s)

Description of models used	Four separate models, varying in terms of number of travel mode binary variables and number of covariates	Four separate models, varying in terms of number of interaction terms and number of covariates	Four separate models, varying in terms of number of transition variables and number of covariates	Twelve separate models, with binary dependent variables representing each of the GHQ12 symptoms

Method of regression analysis	Linear fixed effects	Linear fixed effects	Linear fixed effects	Fixed effects logit

Table provides a summary of the four groups of analyses which were conducted using STATA (version 12.1).

a The 36-point GHQ12 Likert scale, increasing in psychological wellbeing.

In the first three groups of analyses (I–III), Y_it_ represented psychological wellbeing for each individual (i = i….n) for n individuals in the dataset in wave t (1 ≤ t ≤ 18). Linear individual FE models were used, based on the commonly held assumption that once FE are accounted for, the 36-point Likert scale may be considered continuous (rather than ordinal) (Ferrer-i-Carbonell and Frijters, 2004, Roberts et al., 2011). In the fourth group of analyses (IV), Y_it_ represented twelve binary dependent variables used in separate FE logit models of each of the GHQ12 symptoms.

The main exposure of interest was represented by X_k,it_. In each group of analyses (excluding II), models varied in terms of the number of binary variables (k = 1…K for individual i in wave t) depending on how travel mode (analyses I and IV) or travel mode transition (III) was represented (e.g. in the first group of analyses, active travel was first represented by a single binary variable, and then by separate binary variables for walking and cycling). In the second group of analyses (II), following Roberts et al., X_k,it_ in Eq. (1) is replaced by continuous interaction terms of travel time (D, minutes) with travel mode and gender (S):

(2) $$\beta_{1}D_{\mathrm{it}}\;+\;\beta_{k}\left(D_{\mathrm{it}}\;\times \;X_{k,\mathrm{it}}\right)\;+\;\beta_{2}\left(D_{\mathrm{it}}\;\times \;S_{i}\right)$$

In all analyses, Z_j,it_ represented a vector of J covariates (j = 1…J). α_i_ (i = 1…N) was the unobserved individual specific intercept (assumed to be time-invariant and correlated with observed explanatory variables); β and γ were the coefficients, and u_it_ was the error term (assumed to be independent, identically distributed).

Sensitivity analyses explored the impact of excluding groups of individuals with the shortest commutes, as well as observations where participants experienced adverse health states, self-employment and house or job moves.

## Results

### Sample description

Table 2 shows basic descriptive statistics of the sample. There was an even gender split among the 17,985 individuals, the mean age was 39 years, and the mean value of the 36-point GHQ12 scale was 25.29 (with a within-individual standard deviation (SD) of 3.63). Of 102,502 observations where data on wellbeing and travel mode was reported, 73.4% were car travel, 15.8% active travel (of which 3.0% of total observations were cyclists, and 12.8% walkers), and 10.9% public transport (3.3% were rail and 7.6% bus users) (2596 further observations were excluded from the sample due to missing values in the wellbeing variable). Of 75,428 pairs of consecutive waves, maintenance of car travel was most common in 54,727 cases (72.6% of total). Switching occurred between active travel and other modes in 3911 cases (5.2% of total), and between public transport and other modes in 2763 cases (3.7%).

**Table 2:** Descriptive statistics for selected variables and transition probabilities.

	Sample size		Mean values								Number of transitions Total = 75,428^a^		
	N observations (% of total)	n individuals (% used each mode at least once)	Age (s.d.)^c^	Male, %	Couple (including married), %	Commuting time, minutes (s.d.)^c^	Household equivalised income, £ (s.d.)^c^, ^i^	Psychological wellbeing (36-point GHQ12 Likert scale) (s.d.)^c^	Job satisfaction (7 point scale) (s.d.)^c^	Self-employed, %	Car (transition probability)^b^	Active (transition probability)^b^	Public transport (transition probability)^b^
All	102,502^d^ (100%)	17,985 (100%)	39.04 (11.50, 3.59)	50.9%	73.6%	23.41 (20.86, 18.39)	28,843.73 (20,277.46, 15,476.65)	25.29 (4.97, 3.63)	5.38 (1.29, 0.96)	7.8%	57,280 (75.9%)	10,967^e^ (14.5%)	7181^f^ (9.5%)
Car users	75,218 (73.4%)	13,508 (75.1%)	39.62 (11.10, 3.53)	54.8%	76.8%	22.90 (19.66, 12.03)	30,141.22 (19,635.54, 13,101.84)	25.35 (4.89, 3.57)	5.38 (1.27, 0.94)	9.1%	54,727 (96.5%)	1293 (2.3%)	722 (1.3%)
Active travel users	16,140^g^ (15.8%)	5354 (29.8%)	38.39 (12.38, 2.67)	41.1%	66.6%	12.33 (9.91, 4.39)	23,406.79 (22,397.84, 14,344.27)	25.20 (5.11, 3.25)	5.46 (1.33, 0.83)	4.8%	1565 (13.9%)	9,152 (81.4%)	531 (4.7%)
Public transport users	11,144^h^ (10.9%)	3972 (22.1%)	36.07 (12.26, 2.65)	39.4%	59.5%	42.65 (26.29, 12.17)	27,960.52 (19,954.32, 9831.47)	24.97 (5.31, 3.34)	5.28 (1.38, 0.89)	2.9	988 (13.3%)	522 (7.0%)	5928 (79.7%)

Data was collected 1991–2009 in the UK.

a Pairs of individual-specific consecutive waves.

b The final three columns of the table show transition probabilities in which horizontal rows represent travel mode in lagged waves (t − 1) (which add to 100%) and vertical columns represent travel mode in current wave (t).

c s.d. = standard deviation (overall, within individuals).

d After exclusion, first, of the following travel modes: car/van passenger (8714 observations), motorcycle (1201 observations) and underground/metro (1515 observations) (see Methods) and, second, after exclusion of 2596 observations due to missing values in the dependent GHQ12 variable.

e Of which 8791 (11.7% of total) were walkers in time t, and 2176 (2.9%) were cyclists in time t.

f Of which 2375 (3.2%) were railway users in time t, and 4806 (6.4%) were bus users in time t.

g Of which 13,089 (12.8% of total) were walkers and 3051 (3%) were cyclists.

h Of which 3408 (3.3%) were railway users and 7736(7.6%) were bus users.

i Accounting for number of people in the household and the age of children on living standards (see Methods).

### Fixed effects analyses

Table 3 shows results for the first three groups of analyses (I–III), with sensitivity analyses for the first group shown in the Appendix A. Table 4 shows results for the fourth group (IV).

**Table 3:** Results.

Panel I: Fixed effects estimates of the impact of travel mode on psychological wellbeing (higher score = better psychological wellbeing)				
	Minimally adjusted model^a^	Fully adjusted models^b^		
	Model A	Model B	Model C^c^	Model D
	Active travel binary independent variable only		Active travel and public transport binary independent variables	Mode-specific binary independent variables
*Active travel modes*				
Cycling and walking	0.145^⁎^ (0.017)	0.137^⁎^ (0.040)	0.185^⁎⁎^ (0.008)	
Cycling only				0.077 (0.521)
Walking only				0.222^⁎⁎^ (0.004)

*Public transport modes*				
Train, bus and coach			0.195⁎ (0.017)	
Train only				0.161 (0.222)
Bus and coach only				0.216⁎ (0.019)
Observations	101,671^d^	86,065^e^	86,065	86,065
r^2^	0.04	0.08	0.08	0.08



Panel II: Fixed effects estimates of the impact of commuting time and commuting time-travel mode interaction terms on psychological wellbeing (higher score = better psychological wellbeing)				

	Minimally adjusted model^a^	Fully adjusted models^b^		
	Model E	Model F	Model G	Model H

	No travel-mode interaction terms		Non-car interaction terms	Car interaction term only

Time (min)	0.000 (0.996)	− 0.000 (0.933)	− 0.002 (0.214)	0.001 (0.436)
Time × gender	− 0.004^⁎^ (0.039)	− 0.004^⁎^ (0.048)	− 0.004 (0.070)	− 0.004 (0.066)

*Commuting time-active travel*				
Time × walk			0.008⁎ (0.042)	
Time × bike			− 0.001 (0.827)	

*Commuting time-public transport*				
Time × train			0.003 (0.124)	
Time × bus/coach			0.003 (0.160)	

*Commuting time-car*				
Time × car				− 0.003⁎ (0.040)
Observations	109,169	96,222	86,065	86,065
r^2^	0.04	0.08	0.08	0.08


Panel III: Fixed effects estimates of impact of travel mode transitions on psychological wellbeing (higher score = better psychological wellbeing)				

	Minimally adjusted model^a^	Fully adjusted models^b^		

	Model J	Model K	Model L	Model M

	Active travel binary variable only		Active travel and public transport binary variables	Mode-specific binary variables

*Switching to active travel from car travel or public transport*				
Cycling and walking	0.537⁎⁎⁎ (< 0.001)	0.468 (0.001)⁎⁎		

*Switching to active travel from car travel*				
Cycling and walking			0.479⁎⁎ (0.001)	
Cycling				0.168 (0.506)
Walking				0.618⁎⁎⁎(< 0.001)

*Switching to public transport from car travel*				
Train, bus and coach			0.240 (0.206)	
Train				0.266 (0.360)
Bus and coach				0.221 (0.372)
Observations	63,642	56,387	51,305	51,305
r^2^	0.04	0.09	0.09	0.09

Notes to Table 3:

Model A and Model B: Car travel and public transport are in the reference category; Model C, Model D and Model G: Car travel is in the reference category; Model H: Active travel and public transport are in the reference category; Model J and Model K: Maintenance of car travel and maintenance of public transport are in the reference category; Model L and Model M: Maintenance of car travel is in the reference category.

*P*-values shown in parentheses.

Data was collected 1991–2009 in the UK.

⁎ Indicates statistical significance at the p < 0.05 level.

⁎⁎ Indicates statistical significance at the p < 0.01 level.

⁎⁎⁎ Indicates statistical significance at the p < 0.001 level.

a Minimally adjusted models controlled for region, year, age squared, adjusted gross annual household income, number of children and self-assessed health status.

b Fully adjusted models controlled additionally for educational attainment, work hours, neighbourhood characteristics, daily commuting time, job satisfaction and number of previous residences and workplaces.

c Sensitivity analyses for Model C are shown in the Appendix A. The covariates which had a statistically significant impact on wellbeing were: number of children (+ 0.07), being in a couple (including marriage) (+ 0.44), self-assessed health status (+ 2.24 to + 4.10 when compared to poor or worse health), reporting that the participant liked living in their current neighbourhood (+ 0.43), job satisfaction (+ 0.79 per unit change), moving job (+ 0.06), moving house (+ 0.07), and age squared (+).

d An additional 831 observations were excluded from the analysis due to missing values in the adjusted gross annual household income and educational attainment variables.

e 15,606 observations were excluded from Model B, when compared to Model A, due to missing values in the following variables: educational attainment, work hours, neighbourhood characteristics, daily commuting time, job satisfaction and number of previous residences and workplaces.

**Table 4:** Results. Twelve models of the effect of travel mode choice on specific aspects of the GHQ12.

	Constantly under strain	Feelings of being worthless	General unhappiness	Less able to make decisions	Less able to play a useful role	Losing confidence	Lost sleep over worry	Problems overcoming difficulties	Unable toconcentrate	Unable to enjoynormaldaily activities	Unable to face problems	Unhappy/depressed
Public transport	0.889^a^ (0.023)	0.127 (0.204)	0.931 (0.280)	0.934 (0.427)	1.009 (0.911)	1.091 (0.218)	0.872^a^ (0.022)	0.926 (0.254)	0.944 (0.351)	0.981 (0.760)	1.019 (0.812)	0.983 (0.757)

Active travel	0.884⁎ (0.006)	0.958 (0.604)	0.890 (0.052)	0.834^a^ (0.018)	1.054 (0.449)	0.995 (0.941)	0.914 (0.084)	0.911 (0.116)	0.847⁎ (0.003)	0.894^a^ (0.042)	0.916 (0.214)	0.937 (0.188)

Observations	60,855	21,811	42,055	28,298	32,298	36,004	50,633	40,688	47,874	48,572	32,047	53,645

Table shows conditional logit fixed effects estimates of the odds of active travel and public transport users experiencing twelve symptoms of the GHQ12 when compared to car travel.

Dependent variable in each model: 1 = symptoms, 0 = no symptoms.

*P*-values shown in parentheses.

All models control for the same exposure of interest and covariates as Model C (see Table 3).

Data was collected 1991–2009 in the UK.

⁎ Indicates statistical significance at the *p* < 0.05 level after the Bonferroni adjustment for multiple comparisons.

a Indicates statistical significance at the *p* < 0.05 level without adjustment for multiple comparisons (Perneger, 1998).

### (I): Impact of travel mode on wellbeing

In the minimally adjusted model, wellbeing was higher by 0.145 on the 36-point GHQ12 scale when participants used active travel modes compared to car travel or public transport (Model A, Table 3: 95% CI: 0.026 to 0.263). After adjustment for all covariates, a positive association was also found with active travel when compared to car travel (Model C: 0.185, 95% CI: 0.048 to 0.321). Many of the covariates also had a comparable statistically significant impact on the GHQ12 scale. For example, wellbeing was higher by 0.432 (95% CI: 0.306 to 0.557) when participants reported being in a relationship (including marriage) compared to being single. Wellbeing was also higher by 0.434 (95% CI: 0.288 to 0.579) when participants reported that they liked living in their current neighbourhood compared to if they did not (Model C). Due to missing values in the covariates, 831 observations were excluded from the minimally adjusted models and 15,606 were excluded from the fully adjusted models.

Sensitivity analyses (see Appendix A) showed that these results were robust to exclusion of the self-employed (7.76% of observations) (Model C: 0.187, 95% CI: 0.051 to 0.324), between-wave changes in work or home location, and to inclusion of ‘motorcycle’, but not ‘car/van passenger’, in the reference category. Larger effect sizes were identified when chest or breathing difficulties were reported (0.483, 95% CI: 0.062 to 0.901) compared to cases where participants had reported good or better self-assessed health status (0.192, 95% CI: 0.048 to 0.335), and when shortest commute times were excluded (rising from 0.309 to 0.501 for observations where commute times exceeded 10 and 30 min respectively).

A positive wellbeing effect was also found with public transport (Model C, Table 3: 0.195, 95% CI: 0.035 to 0.355), and with walking (Model D: 0.222) and bus/coach travel (0.216), when compared to car travel.

### (II): Impact of travel time on wellbeing

Positive associations were identified between time spent walking (per ten minute change) and wellbeing (with car travel in the reference category) (Model G: a 10 minute increase in walking was associated with an increase in the GHQ12 of 0.083). Negative associations were identified between time spent driving and wellbeing (with all other travel modes in the reference category) (Model H: − 0.033). A negative association was also found between travel time and wellbeing for women in the models that did not include the travel mode interaction terms (Models E and F).

### (III): Impact of switching to more active travel modes on wellbeing

In the minimally adjusted model, switching from car travel or public transport to active travel was associated with an improvement in wellbeing of 0.537 on the GHQ12 scale (during the wave in which the switching took place) when compared to maintaining car travel or public transport (Model J: 95% CI: 0.199 to 0.758). After full adjustment, switching from car to active travel (Model L), or from car to walking (Model M: 0.618, 95% CI: 0.284 to 0.952), was also associated with improvement in wellbeing when compared to maintaining car travel.

### (IV): Impact of travel mode on specific aspects of wellbeing

The likelihood of reporting being constantly under strain or unable to concentrate was at least 13% higher when participants used car travel, compared to active travel, after Bonferroni correction for multiple comparisons (Table 4: the odds ratios for experiencing these symptoms of 0.884 and 0.847 were statistically significant for active travel users when compared to car travel) (Perneger, 1998).

## Discussion

### Active travel and wellbeing

Our main observation of a positive association between active commuting and wellbeing was supported by four distinct groups of analyses. Causal inference was better supported, when compared to existing cross-sectional studies, by using the FE framework. We also accounted for potential time-varying confounding variables, including job satisfaction, residence, workplace and health, and identified a specific ‘switching effect’ in addition to statistical associations between travel mode and wellbeing. Furthermore, the commuting time analyses showed a positive relationship between time spent walking and wellbeing which, together with the observed increased effect sizes as participants with shorter commutes were progressively excluded from the first group of analyses, indicate a dose–response relationship.

Our main findings contrast with two recent cross-sectional studies of commuter behaviour in the UK which did not identify any statistically significant positive association between active commuting and wellbeing (ONS, 2014), or between time spent in active commuting and wellbeing (Humphreys et al., 2013, Office for National Statistics (ONS), 2014). In one of these cross-sectional studies, published by the Office for National Statistics (ONS), statistically significant negative associations were identified between walking (or cycling for journeys of 16–30 min) and most aspects of psychological wellbeing when compared to car travel. Nonetheless, our findings are consistent with other studies (Hamer et al., 2009), including randomised studies of exercise interventions (Dunn et al., 2005, Gusi et al., 2008), which identified positive associations between some aspects of wellbeing and physical activity in other domains.

### Public transport and wellbeing

The positive association observed between wellbeing and public transport when compared to car travel was of a comparable magnitude to that observed between wellbeing and active travel. This finding contrasts with the cross-sectional ONS study that identified statistically significant negative associations between commuting by bus or rail (for journeys of at least 30 min) and all or some aspects of wellbeing (when compared to shorter journeys by any mode) (ONS, 2014). A partial explanation for our finding could be that public transport journeys typically feature physical activity when accessing bus stops or railway stations (Besser and Dannenberg, 2005, Edwards, 2008, Laverty et al., 2013, MacDonald et al., 2010, Morabia et al., 2010, Rissel et al., 2012). However, there are other explanatory factors that may well have both positive and negative effects. For instance, public transport may provide important opportunities for catching up with work or friends, whilst crowded carriages may soon become unpleasant (Abou-Zeid et al., 2012, Eriksson et al., 2013, Ettema et al., 2012, Jain and Lyons, 2008, Olsson et al., 2013, Roberts et al., 2011, Stutzer and Frey, 2008).

### Travel mode choices are more important than travel time

The negative association observed between wellbeing and travel time amongst women (Model F: a 10 minute increase in commuting time using any travel mode was associated with a reduction in the 36-point GHQ12 Likert scale of 0.040) and car drivers (Model H: a 10 minute increase reduced the GHQ12 by 0.033) is broadly consistent with existing studies (a 10 minute increase reduced the GHQ12 by 0.055 in Roberts et al.) (Office for National Statistics (ONS), 2014, Roberts et al., 2011). Nevertheless, given that these are small effect sizes and a similar positive relationship was identified between time spent walking and wellbeing (Model G: a 10 minute increase in walking increased the GHQ12 by 0.083), we conclude that the potential benefits available to car drivers if they switched to active travel (Model L: switching was associated with an increase in the GHQ12 of 0.479), and walking in particular (Model M: 0.618), exceed any potential benefits associated with reducing commuting time. Besides, only a small journey time mean and variance was observed amongst car drivers in the sample (mean = 22.9 min, within-individual SD = 12.03).

Together, these results appear to suggest that avoiding car driving may be beneficial to wellbeing. This view complements existing evidence of a negative association between driving and physical health (Frank et al., 2004, Jacobson et al., 2011), and is consistent with the hypothesis that car driving (a non-passive travel mode that requires constant concentration (Roberts et al., 2011)) can give rise to boredom (Gatersleben and Uzzell, 2007), social isolation and stress (Gottholmseder et al., 2009, Office for National Statistics (ONS), 2014, Roberts et al., 2011). However, this view is also consistent with the hypothesis that intrinsic enjoyment is gained from the exercise or relaxation associated with active travel (Gatersleben and Uzzell, 2007, Guell and Ogilvie, 2013, Olsson et al., 2013). Hence despite being (to our knowledge) the first longitudinal study to identify associations between travel mode choices and specific aspects of wellbeing included in the GHQ12, more research is necessary on the exact causal mechanism by which car driving appears to impact negatively on wellbeing.

### Study limitations

Whilst the BHPS provided significantly larger sample sizes than would be available in primary intervention studies, relatively few participants were ever active travel users. Cycling and rail travel were especially rare, limiting the study of mode-specific effects in these cases. Although our results could be biased by the small number of missing values in the covariates, since these may not be missing at random, our results in the fully adjusted models were consistent with those in the minimally adjusted models which included only those covariates that had the fewest missing values. Richer data relating to unobserved features of the built environment, or physical activity behaviour, could also have supported more detailed study of causal mechanisms or differential effects between individuals and contexts. For example, active commuting could be more beneficial in natural environments, when compared to urban environments (Bostock, 2001, Mitchell, 2013) where other factors (e.g. the perceived security or safety of car travel (Guell and Ogilvie, 2013)) may dominate, and walking pace could be more informative than time spent walking (Tanasescu et al., 2002). Considering the relatively large sample variance of the wellbeing variable (SD = 3.63), the observed effect of switching travel mode was also relatively small. Hence complementary evidence on physical activity (Sahlqvist et al., 2013) and physical health outcomes (Flint et al., 2014, Wanner et al., 2012) should be considered when assessing the potential population-level impact of behaviour change interventions. Further, this study does not explore the reasons for, or the feasibility of, switching travel modes (Clark et al., forthcoming). Our results complement existing UK studies; however, in other countries cultural factors may have an important influence on attitudes towards different travel modes and the associated impact on wellbeing. Compared to the US, for example, where active travel and public transport use is not so mainstream and communities have been designed with little consideration for these modes (Sallis et al., 2004), European countries are said to benefit from unbroken traditions of utilitarian cycling, better facilities and more supportive road traffic regulations for walkers and cyclists, as well as less corporate power in the transport sector (Buehler, 2011, Pucher et al., 1999).

## Conclusion

In addition to potential physical health benefits, the positive psychological wellbeing effects identified in this study should be considered in cost–benefit assessments of interventions seeking to promote active travel.

## Conflict of interest statement

The authors declare that there are no conflicts of interest.

## Figures and Tables

**Table:** 

		Subgroup analyses								Inclusion of additional travel modes in the travel mode categories	
	Model C	(a)	(b)	(c)	(d)	(e)	(f)	(g)	(h)	(i)	(j)
Public transport	0.195⁎	0.057⁎	0.208⁎	0.200⁎	0.157	0.365	0.286⁎⁎	0.392⁎⁎⁎	0.513⁎⁎⁎	0.149	0.086
Active travel	0.185⁎⁎	0.195⁎	0.200⁎⁎	0.187⁎⁎	0.192⁎⁎	0.483⁎	0.309⁎⁎	0.337⁎	0.501⁎	0.159⁎	0.095
Observations	86,065	61,488	75,007	85,801	68,845	13,769	56,530	44,119	31,877	88,382	95,677
r^2^	0.08	0.08	0.08	0.08	0.08	0.10	0.09	0.09	0.10	0.08	0.010

Model C (see Table 3) is shown for comparison since the same covariates were used in models (a) to (j).

Car travel was in the reference category.

Data was collected 1991–2009 in the UK.

⁎ Indicates statistical significance at the *p* < 0.05 level.

⁎⁎ Indicates statistical significance at the *p* < 0.01 level.

⁎⁎⁎ Indicates statistical significance at the *p* < 0.001 level.

## Appendix A Sensitivity analyses

Using Model C, the fully adjusted model of travel mode on psychological wellbeing with active travel and public transport binary variables, this section presents sensitivity analyses as follows:

### Subgroup analyses

(a) Excluding between-wave transitions where participants moved job, and (b) excluding between-wave transitions where participants moved residence, since these changes may impact on wellbeing. (c) Excluding observations where participants reported being self-employed, since travel patterns may vary when compared to the majority of workers who are employed. (d) Excluding observations where participants reported fair or worse self-assessed health, and (e) including only observations where participants reported having chest or breathing difficulties, since these may be a proxy for overall fitness and/or potential confounding variables. Including only observations where commuting time was (f) greater than 10 min, (g) greater than 20 min, and (h) greater than 30 min, in order to study the dose–response relationship between physical activity and wellbeing.

### Inclusion of additional travel modes in the travel mode categories

(i) Inclusion of underground in the public transport group, and motorcycle in the 'car travel' reference category, and (j) inclusion of underground in the 'public transport' category, and motorcycle and car/van passenger in the 'car travel' reference catergory. These travel modes had been excluded from the main analyses due to small sample sizes.
